# Barreras y facilitadores a la implementación de la telemedicina en las Américas

**DOI:** 10.26633/RPSP.2021.131

**Published:** 2021-10-18

**Authors:** Sebastian Garcia Saiso, Myrna C. Marti, Victoria Malek Pascha, Adrian Pacheco, Daniel Luna, Fernando Plazzotta, Jennifer Nelson, Luis Tejerina, Alexandre Bagolle, Maria Celeste Savignano, Analia Baum, Pablo J. Orefice, Ana Estela Haddad, Luiz Ary Messina, Paulo Lopes, Francesc Saigí Rubió, Daniel Otzoy, Walter H. Curioso, Antonio Luna, Felipe Mejia Medina, Janine Sommer, Paula Otero, Fernán González Bernaldo De Quiros, Marcelo D’Agostino

**Affiliations:** 1 Organización Panamericana de la Salud Washington D.C. Estados Unidos Organización Panamericana de la Salud, Washington D.C., Estados Unidos.; 2 Consultora internacional Argentina Consultora internacional, Argentina; 3 CENETEC Ciudad de México México CENETEC, Ciudad de México. México; 4 Hospital Italiano de Buenos Aires Ciudad Autónoma de Buenos Aires Argentina Hospital Italiano de Buenos Aires, Ciudad Autónoma de Buenos Aires, Argentina; 5 Banco Interamericano de Desarrollo Washington D.C. Estados Unidos Banco Interamericano de Desarrollo, Washington D.C., Estados Unidos; 6 Hospital de Pediatría "Prof Dr. Juan P. Garrahan" Ciudad Autónoma de Buenos Aires Argentina Hospital de Pediatría "Prof Dr. Juan P. Garrahan", Ciudad Autónoma de Buenos Aires, Argentina; 7 Ministerio de Salud de la Ciudad Autónoma de Buenos Aires Argentina Ministerio de Salud de la Ciudad Autónoma de Buenos Aires, Argentina; 8 Salud.uy Montevideo Uruguay Salud.uy, Montevideo, Uruguay; 9 Universidad de Sao Paulo San Pablo Brasil Universidad de Sao Paulo, San Pablo, Brasil; 10 Rede Nacional de Ensino e Pesquisa Rio de Janeiro Brasil Rede Nacional de Ensino e Pesquisa, Rio de Janeiro, Brasil; 11 Universitat Oberta de Catalunya Barcelona España Universitat Oberta de Catalunya, Barcelona, España; 12 Red Centroamericana de Informática en Salud Guatemala Red Centroamericana de Informática en Salud, Guatemala, Guatemala; 13 Universidad Continental Lima Perú Universidad Continental, Lima, Perú.; 14 Consultor internacional Colombia Consultor internacional, Colombia

**Keywords:** Acceso a la información, salud pública, práctica de salud pública, planificación en salud, telemedicina, Access to information, public health, public health practice, health planning, telemedicine, Acesso à informação, saúde pública, prática de saúde pública, planejamento em saúde, telemedicina

## Abstract

Con millones de personas en el mundo en situación de distanciamiento físico por el COVID-19, las tecnologías de la información y comunicaciones (TICs) se han posicionado como uno de los medios principales de interacción y colaboración. Ya al inicio de este milenio se empezaban a mencionar las siguientes ventajas: mayor acceso a la información y a la prestación de servicios; fortalecimiento educativo; control de calidad de los programas de detección y reducción de los costos de la atención de en salud. Sin embargo, entre las principales barreras de adopción de la telemedicina se encuentran las de índole: tecnológicas; humanas y sociales; psico-sociales y antropológicas; de Gobernanza y económicas. En estos 20 años se logró un aumento en los recursos y capacidad técnica, una mejora en la educación digital, un empoderamiento del paciente en su tratamiento y un mayor interés público en esta área. En especial se considera exitosa la conformación de equipos interdisciplinarios, las redes académicas y profesionales y las consultas médicas virtuales. Después de revisar el estado de la telemedicina en la Región de las Américas, los autores recomiendan adoptar medidas urgentes para poner en práctica políticas y programas nacionales de telemedicina, incluyendo el marco normativo y presupuesto necesario, cuya implementación se realice de manera integral e interoperable y que se sustente de redes académicas, de colaboración e instituciones especializadas. Dichas políticas deben generar un contexto habilitante que den sostenibilidad al avance logrado, considerando los aspectos mencionados en las posibles barreras.

## Abstract (en)

With millions of people in the world in situations of physical distancing because of COVID-19, information and communication technology (ICT) has become as one of the principal means of interaction and collaboration. The following advantages of ICT have been cited since the start of the new millennium: increased access to information and service delivery, educational strengthening, quality control of screening programs, and reduction of health care costs. In the case of telemedicine, however, a number of barriers—especially technological, human and social, psychosocial, anthropological, economic, and governance-related—have stood in the way of its adoption. The past 20 years have seen an increase in the availability of resources and technical capacity, improvements in digital education, empowerment of patients regarding their treatment, and increased public interest in this area. Successes have included the use of interdisciplinary teams, academic and professional networking, and virtual medical consultations. After reviewing the state of telemedicine in the Region of the Americas, the authors recommend the urgent adoption of measures aimed at implementing national telemedicine policies and programs, including a regulatory framework and adequate funding. Implementation of the measures should be integrated and interoperable and include the support of academic networks and the collaboration of specialized institutions. The policies should generate an enabling context that ensures sustainability of the progress achieved, bearing in mind the possible barriers mentioned.

## RESUMO

Com milhões de pessoas no mundo em situação de distanciamento físico devido à COVID-19, as tecnologias da informação e comunicação (TICs) se enquadraram como um dos meios principais de interação e colaboração. Já no início deste milênio, começaram a ser mencionadas as seguintes vantagens: maior acesso à informação e à prestação de serviços; fortalecimento da educação; controle de qualidade dos programas de detecção e redução dos custos na atenção à saúde. No entanto, entre as principais barreiras de adoção da telemedicina se encontram as de caráter: tecnológico; humano e social; psicossocial e antropológico; de governança e econômico. Nestes 20 anos, houve um aumento nos recursos e na capacidade técnica, uma melhora na educação digital, um empoderamento do paciente em seu tratamento e um maior interesse público nessa área. Em especial, são consideradas bem-sucedidas a constituição de equipes interdisciplinares e as redes acadêmicas e profissionais, e as consultas médicas virtuais. Após revisar o estado da telemedicina na Região das Américas, os autores recomendam a adoção de medidas urgentes para implementar políticas e programas nacionais de telemedicina, incluindo o marco normativo e o orçamento necessário. Essa implementação deve ser realizada de maneira integral e interoperável e sustentada por redes acadêmicas, de parceria e instituições especializadas. Tais políticas devem gerar um contexto favorável, dando sustentabilidade ao avanço obtido e considerando os aspectos mencionados nas possíveis barreiras.

El primer registro en la historia de uso de tecnologías de la información y comunicaciones (TICs) para transmitir una prestación médica fue en 1899 cuando el Dr. Alejandro Posadas utilizó el cinematógrafo para filmar una cirugía de quiste hidatídico y que posteriormente se transmitió a diversas localidades de la República Argentina (1). Hoy, con millones de personas en el mundo en situación de distanciamiento físico por el COVID-19, cierres de fronteras, trabajo y educación a distancia, las TICs se han posicionado como uno de los medios principales por el cual individuos, gobiernos e instituciones de salud trabajan, interactúan, comparten información, colaboran, generan conocimiento, y se comunican (2). Entre aquella acción innovadora de 1899 y el presente hubo una serie de desarrollos y políticas que sentaron las bases y el conocimiento necesario para que la telemedicina pueda ser una parte integral de los servicios de salud en la Región de las Américas. La pandemia del COVID-19 ha demostrado la importancia de la telemedicina y la telesalud para sustentar la continuidad de los servicios asistenciales, especialmente de aquellas enfermedades crónicas no transmisibles y condiciones de salud mental (2). El presente documento tiene como objetivo presentar un recorrido por los últimos veinte años de la telemedicina, identificando sus objetivos, logros, oportunidades de mejora, aprendizajes y las lecciones aprendidas en durante la pandemia de COVID-19 en la temática. La información aquí incluida es el resultado del análisis de un grupo de expertos en la temática, trabajado a través una convocatoria en línea de creación asincrónica sobre unas guías de preguntas. Se cubrieron aspectos académicos, de políticas públicas, normativos, asistencia hospitalaria y redes de conocimiento tanto del ámbito público como privado, con especial énfasis en la Región de las Américas.

## ¿QUÉ SE PRETENDÍA LOGRAR DOS DÉCADAS ATRÁS?

Ya al inicio de este milenio, algunos estudios vislumbraban los posibles beneficios de la telemedicina para facilitar el desarrollo de políticas públicas y mejoras a los sistemas de salud. Entre las ventajas se mencionaban: acceso a la información, acceso a la prestación de servicios -tradicionalmente relegados por la presencialidad-, fortalecimiento educativo, control de calidad de los programas de detección y reducción de los costos de la atención de en salud (3). Asimismo, ya se planteaba que el desarrollo de las nuevas tecnologías posibilitaría el acceso de las personas a mejores servicios de salud en general. Sin embargo, también se planteaban preocupaciones, que pueden haber oficiado como barreras para la aceleración de la telemedicina, tales como una posible ruptura de la relación médico-paciente y entre profesionales, la pérdida de la calidad de la atención, consideraciones regulatorias y cuidados respecto la exactitud del registro en la historia clínica (3,4).

## ¿QUÉ FUE LO QUE REALMENTE SE LOGRÓ?

En la Región de las Américas los avances técnicos, el desarrollo de herramientas, publicaciones y materiales educativos son vastos y diversos y se consideran como uno de los principales logros. Como una consecuencia directa se destacan avances en los siguientes aspectos:

**Recursos y Capacidad técnica:** en aquellos países de la Región con mayor aceptación de la telemedicina, se ha conseguido pasar de la financiación internacional a la gubernamental, permitiendo que las instituciones puedan brindar servicios de telemedicina de manera acelerada y segura como la llamada “nueva normalidad” está exigiendo (5). Entre 2005 y 2016, en aquellos países donde se ha invertido en la asistencia remota pública se han alcanzado resultados sorprendentes con tasas de 70-80% de transferencias evitadas de pacientes a centros de salud especializados o de mayor complejidad; ahorros de 10-15% en el presupuesto municipal de salud y más de 10 millones de segundas opiniones en electrocardiogramas y exámenes de imágenes (6,7).

**Educación:** se han desarrollado redes universitarias de telemedicina que no solo facilitaron y fortalecieron la conexión entre las instituciones de enseñanza sino también entre servicios sanitarios de sistemas públicos; articulando la gestión de los departamentos de salud estatales y municipales para fomentar una cultura de atención a distancia. Como ejemplo de esto, en Brasil, la iniciativa se originó del Ministerio de Salud y supuso una integración entre el sistema único de salud y las universidades públicas. A nivel hospitalario, la adopción de la Telemedicina es un 30% mayor en los hospitales públicos frente a los privados (5,8–10). Además, existe una noción generalizada en los profesionales de la salud de que la incorporación de TICs como apoyo a su práctica, tiene beneficios considerables.

**Empoderamiento del paciente:** se logró una participación de los pacientes por primera vez mediada por tecnologías digitales para el cuidado de salud, en especial en la prevención de la trasmisión a través del monitoreo de casos sospechosos durante la pandemia de COVID-19.

**Interés público:** el avance de salud digital en muchos países de la Región ha posibilitado que los Ministerios de Salud incluyeran en sus agendas aspectos relacionados a la telemedicina como elemento de apoyo para procesos, normativas, políticas y cambios administrativos importantes que ya permiten una adopción de las nuevas tecnologías digitales (8,11,12).

## ¿QUÉ FUE CONSIDERADO EXITOSO Y POR QUÉ?

La conformación de equipos interdisciplinarios, que incluyen profesionales de la salud, administrativos, pacientes, personal técnico, especialistas en tecnología y seguridad informática. Este gran capital social ganado, está siendo clave para sortear las brechas digitales que se han presentado. Se destaca que la verdadera innovación en servicios de salud no es la tecnología por sí misma, sino la nueva forma de articulación de los actores, la transformación de los procesos atenta a los escenarios cambiantes de nuevas expectativas y necesidades. Es importante comprender que la implementación de un programa de telemedicina encuentra su mayor desafío en el cambio cultural, entonces tiene mucho valor la conformación de un equipo interdisciplinario que pueda llevar estrategias de gestión del cambio y acompañar a todos los actores involucrados.

Las redes académicas y profesionales, tales como las universitarias, académicas o de investigación, entre otras, fueron y seguirán siendo uno de los pilares para el desarrollo e implementación de servicios de telemedicina, propiciando espacios de intercambio de información y de gestión del conocimiento. Estas redes resultaron fundamentales para la creación de capacidades a lo largo de todo el continente, que hoy cuenta con un colectivo listo para la acción, a la velocidad que requieren las emergencias sanitarias y las medidas de salud pública (13–15).

La experiencia de más de 14 años de estas redes se ha consolidado en un gran número de proyectos y servicios públicos de capacitación diaria en diversas áreas en el ámbito nacional, provincial y municipal. Estos incluyen: proyectos piloto, mejora de la logística de las líneas de espera, ampliación de la oferta de servicios de salud, expansión del acceso a los servicios, perfeccionamiento de la calidad de los servicios brindados, evidencia científica, desarrollos y puesta en marcha un sin número de aplicaciones, plataformas, *startups*, parques tecnológicos en salud, protocolos, reglamentaciones, leyes y normas. Esto está resultando en la inserción gradual de todas las profesiones de salud en las TICs, permitiendo a los ingenieros y científicos de computación trabajar de forma coordinada con profesionales de salud y la población, beneficiándose mutuamente (16).

Las consultas médicas virtuales o “teleconsultas**”** se convirtieron en una estrategia de atención sanitaria, para mejorar el acceso y la equidad en salud, reduciendo la circulación de las personas y facilitando la continuidad asistencial de los tratamientos, sobre todo a los grupos más vulnerables. Además, el uso de medios virtuales para procesos de segunda opinión formativa fue muy exitoso en varios países de la región. Es importante mencionar la influencia de las nuevas TICs en las transformaciones de hábitos sociales y culturales ya que han expuesto una dinámica diferente que juegan a favor de la adopción de esta forma de prestación de servicios de salud (17,18).

## ¿QUÉ PODRÍA HABERSE HECHO MEJOR Y POR QUÉ?

La mayoría de las intervenciones en telemedicina, han sido desarrolladas con un enfoque mayormente técnico, asumiendo que los actores claves y las partes interesadas lo aceptarían “por defecto”. Este enfoque ha, muchas veces, subestimado temas relevantes como la alfabetización digital, la creación del talento digital y la cultura. Muchos abordajes han sido desde la práctica tradicional de la medicina, prevaleciendo la perspectiva de los profesionales de la salud o los prestadores de servicios y poco la perspectiva de los pacientes, principalmente aquellos desconectados o menos -o no- digitalmente alfabetizados (19,20). En un futuro, se deberá poner mayor atención a aspectos como la usabilidad de aplicativos, la portabilidad (foco en dispositivos móviles, con funcionalidades integradas) y los flujos de trabajo asistenciales generando experiencias digitales pre y post presenciales. Ante la urgencia de la pandemia del COVID-19, se ha primado en la simpleza de la tecnología y no lo correcto ni seguro; por lo cual el desafío a futuro será alcanzar nuevos y mejores niveles de buen uso de la telemedicina.

También, la definición de modelos de sostenibilidad financiera es uno de los aspectos claves a mejorar ya que, al no haber claridad sobre los temas de pagos o reembolsos por telemedicina, las iniciativas no encuentran su régimen de sostenibilidad y muchas se quedan a nivel de piloto o proyecto de baja escala y temporalidad. Se ha observado la ausencia de legislación y normativa adecuada especialmente en países donde las asociaciones de profesionales de la salud decidieron no acompañar y hasta boicotear la implementación de telemedicina fundamentando la resistencia al cambio en aspectos vinculados al “acto médico” (8,9,11,12).

Aunque fue claro el camino para la incorporación sostenida de la telemedicina, la insistencia de incorporar tecnologías sin el respaldo de proyectos estructurados bien definidos, sin interoperabilidad, y de corto plazo predominó en varios países de la Región. Tomarse tiempo para pensar, evaluar y determinar un plan a largo plazo antes de la incorporación de la tecnología, fue poco común entre las estrategias nacionales y regionales. A pesar de contar con documentos y apoyo de organizaciones internacionales, la prisa por obtener resultados inmediatos fue en apariencia más importante que fortalecer las bases y fundamentos de un plan sostenible a largo plazo para la implementación y mantenimiento de programas de telemedicina que respondieran a cualquier cambio de ideología, gestión administrativa, mercado, regulación o capacidades del personal de salud. Esta situación se vio facilitada por la limitada evidencia cuantitativa con metodologías de medición de impacto rigurosas que permitan medir los resultados de las intervenciones de telesalud, especialmente en lo que respecta a la calidad de los servicios y los indicadores de salud de los pacientes.

## ¿QUÉ PODEMOS APRENDER DEL CAMINO RECORRIDO EN TELEMEDICINA?

Iniciativas de esta dimensión social, cultural, técnica, política y económica son imposibles de lograr sin el apoyo gubernamental, un enfoque intersectorial y con acciones transdisciplinarias. El desarrollo de marcos técnicos de acción basados en estándares internacionales, lograr trabajadores de la salud capacitados y usuarios de salud alfabetizados digitalmente deben ser considerados como los principales factores críticos de éxito. Una gobernanza robusta requiere de la incorporación de las redes especializadas y un conjunto de expertos para el desarrollo de planes de implementación, facilitando información, experiencias, capacitación, seguimiento y evaluación de programas, generando acciones de contención ante cambios inesperados. Los actores involucrados podrían documentar los procesos de trabajo desde la macro a la micro-gestión a través de guías de práctica clínica, políticas, procedimientos y documentos similares, para que quienes lideran los equipos trabajen con un entendimiento compartido y oficial en los programas de telemedicina definidos. Resulta clave entonces entender el nivel de madurez de un establecimiento o de una red para calibrar las intervenciones de telesalud de forma coherente con el mismo. Es necesario resaltar, la importancia de la utilización de estándares internacionales y las mejores prácticas reconocidas para que los interesados diseñen, documenten, implementen y monitoreen procesos eficientes y eficaces.

## ¿QUÉ HICIMOS BIEN QUÉ TENEMOS QUE “PROTEGER” Y PROMOVER INTENSAMENTE, O DE LO CONTRARIO SE OLVIDARÁ?

Se han establecido bases sólidas de redes de apoyo interinstitucional, multidisciplinario y con apoyo de la comunidad internacional. Poder generar evidencia a través de la investigación en implementación (I+I) es fundamental para que este nuevo interés en las TICs no se desvanezca con el retorno a la rutina y la “nueva normalidad” (19). De esta manera se puede comprender qué, cómo y por qué una intervención funciona en el mundo real, si se respetan las políticas, planes y programas informados en la evidencia empírica -cuando la haya- o buenas prácticas en innovación, principios éticos y de seguridad informática; y evaluar alternativas u opciones que permitan mejorar la implementación de estas intervenciones. De igual importancia es incluir estudios de costos y costo-efectividad de estos programas para justificar su inversión e implementación a escala.

**CUADRO 1.** Principales barreras identificadas para la adopción de la Telemedicina.

**Tecnológicas**	**Humanas y Sociales**	**Psico-sociales y antropológicas**	**Gobernanza**	**Económicas**
Estándares y certificación	Privacidad	Brecha digital (Uso y acceso)	Licenciamiento y matriculación	Falta de datos y metodologías de evaluación
Infraestructura de TIC adecuadas	Seguridad	Cultura organizacional	Vacíos, grises y colisiones normativas.	Financiamiento de servicios
Interoperabilidad	Integridad	Capacitación de profesionales	Ley y reglamentación	
	Confidencialidad	Investigación vs necesidades reales		
		Formación de estudiantes		N/A

A pesar de contar solo con resultados preliminares y poco material para el desarrollo de la gestión de conocimiento, se pudieron establecer grupos de interés y redes de colaboración que han sido la pieza fundamental en el desarrollo de la telemedicina en la región (20). Es importante mantener el conocimiento adquirido y dialogar con aquellos que tienen más experiencias para evitar la duplicidad de esfuerzos tácticos, operativos y estratégicos aún en los mismos sitios. La conformación de redes de profesionales que puedan compartir sus experiencias, las barreras que encontraron, cómo las sortearon, sobre todo en aquellos sistemas de salud dónde esta modalidad de atención es novedosa es vital, así como fortalecer las redes de colaboración estableciendo mejores estrategias de divulgación, difusión y apoyo a las instituciones y organizaciones.

La facultad de crear nuevo conocimiento aplicable resulta posible si se movilizan velozmente los eslabones de la "cadena de conocimiento”, para ello es necesario considerar lo siguiente: a) conciencia Interna: es la capacidad de las organizaciones para realizar un permanente testeo de sus competencias críticas, produciendo un "abandono organizado" de aquella producción que ya no se ajusta a las condiciones externas; b) capacidad de respuesta interna: es la aptitud para transformar nuestro conocimiento del entorno en acciones concretas que posibiliten traducir nuestras competencias en productos y servicios. Esta situación puede ser difícil de alcanzar ya que, por lo general, se conoce o intuye lo que los usuarios demandan, pero se carece de la capacidad para articular las acciones internas de respuesta; c) capacidad de respuesta externa: consiste en ofrecer acertadamente lo que el usuario demanda y anticiparse a los requerimientos del futuro; y d) conciencia externa: es la capacidad para comprender las tendencias y adecuar nuestra acción a ellas, se alimenta muy especialmente de la inteligencia estratégica y de las relaciones.

## ¿QUÉ DEBEMOS HACER AHORA EN LA LLAMADA “NUEVA NORMALIDAD”?

Mientras algunos países han flexibilizado las regulaciones durante la pandemia, otros siguen con un proceso de adopción más lento. El fortalecimiento de la gobernanza es prioritario, asegurando instrumentos normativos adecuados y estrategias de gestión del cambio para que los servicios de telemedicina sean parte de los servicios de salud (8,11,12). Por ello las asociaciones profesionales deben ser parte integral de este nuevo enfoque y que todos, sin excepción, contribuyan para que sea algo normal y al alcance de todos los habitantes. Es fundamental fomentar mayores espacios de interacción entre la academia y los sectores del gobierno; y eliminar las principales barreras, como se indican en el cuadro 1 que hoy existen en el ámbito tecnológico, organizacional, social, cultural, político, legal y económico.

La incertidumbre provocada por el COVID-19 ha generado el desarrollo de aplicaciones digitales con la intención de brindar soluciones al personal de salud y la ciudadanía, lo que ha desbordado el alcance de las normativas y regulaciones en el área. Es necesario estructurar planes de telemedicina para operar en escenarios disruptivos, capaces de romper paradigmas de atención, considerando todos los escenarios posibles, dentro  de contextos que exceden a las instalaciones y los profesionales de la salud, garantizando niveles de calidad y manteniendo principios bioéticos tan evocados y necesarios dentro del proceso de atención médica.

Se requiere incorporar nuevos actores que proporcionen información para la toma de decisiones, generar una cultura que permita el aprovechamiento de la infraestructura tecnológica ya instalada y crear capacidades para evaluar y discernir entre aquellos procesos plausibles de llevar a cabo mediante el uso de la tecnología y aquellos donde la tecnología sólo será un apoyo.

Con respecto a la sostenibilidad futura es necesario aplicar metodologías de I+I para fortalecer la eficiencia y capacidad de respuesta de los sistemas y servicios de salud y mejorar su impacto en la equidad y nivel de salud de la población.

## ¿CÓMO SE PODRÍAN CONSOLIDAR LAS LECCIONES APRENDIDAS DEL PASADO?

En el cuadro 2 se expone la situación actual de la telemedicina en la región y las lecciones aprendidas durante los últimos veinte años y en particular durante la pandemia del COVID-19

Para el futuro va a ser clave incorporar antes la mirada del paciente y proveedor, qué recursos tecnológicos tiene o qué canales de comunicación usa habitualmente. El mayor aprendizaje recae en la necesidad de comprender que los desafíos en la transformación digital son más humanos, culturales y organizacionales que técnicos, y que el rol de los que lideran estas implementaciones está principalmente en poder articular las necesidades de todos los actores involucrados; que en salud siempre son muchos y con intereses diversos.

Después de revisar el estado de la telemedicina en la Región de las Américas, los autores recomiendan adoptar medidas urgentes para poner en práctica políticas y programas nacionales de telemedicina, incluyendo el marco normativo y estratégico y presupuesto necesario, cuya implementación se realice de manera integral e interoperable y que se sustente de redes académicas, de colaboración e instituciones especializadas. Dichas políticas deben generar un contexto habilitante que den sostenibilidad al avance logrado, considerando los aspectos mencionados en las posibles barreras.

**CUADRO 2.** Comparación de la situación resaltada en pandemia y la consecuente lección aprendida

Situación Actual	Lección Aprendida
1) Existencia de suficientes experiencias implementadas y un gran número, documentadas. Estas pueden servir de base y referencia para guiar procesos actuales o futuros de manera costo-efectiva y sostenible (20,21) El conocimiento puede provenir de fuentes internas o externas, desarrollándolo o adquiriéndolo. Este nunca es producto de la actividad de una organización que trabaja aislada.	Focalizar los esfuerzos en la Implementación. Es clave un marco de cooperación y colaboración entre instituciones y profesionales que hayan sido parte de la implementación, sea con resultados positivos o negativos. El trabajo en grupo, la cooperación entre las instituciones en modelos flexibles y las posibilidades de trabajo virtual, hacen del intercambio y distribución del conocimiento una cuestión prioritaria.
2) La tecnología permitió descubrir y conectar a las personas con el conocimiento técnico. Para la memoria institucional, si bien la tecnología no reemplaza el valor del conocimiento tácito intercambiado mediante el contacto humano directo, facilita la localización de las personas con el conocimiento requerido y la creación de redes entre ellas.	La transformación del mundo cotidiano es más función del comportamiento y cambio en la cultura, que de la tecnología. Sin dudas la conectividad múltiple eleva exponencialmente los canales de comunicación y las posibilidades de establecer nodos para enlazar conocimiento, así como favorece la cultura del conocimiento compartido.
3) Los pacientes se empoderaron forzosamente por esta situación excepcional. Se ha acelerado la posibilidad de que el paciente asuma un rol más activo en el control de su propia salud y así evitar interrupciones en tratamientos provocados por las medidas de distanciamiento físico. Asimismo, la participación del paciente se volvió imprescindible, poder evaluar cómo es nuestra población usuaria, qué recursos tiene y qué canales de comunicación utiliza usualmente.	Incorporar el empoderamiento del paciente a la nueva normalidad. La asistencia, las interconsultas entre profesionales y la capacitación médica por medios virtuales debería pasar a ser una opción permanente y complementaria que ofrecen los servicios de salud. Asimismo, deben estar respaldadas por leyes, presupuestos, reglamentos y códigos de ética de colegios profesionales y pasar a ser parte de las decisiones individuales y clínicas asistenciales.
4) La prestación de servicios de telemedicina es parte de la mirada de salud como un derecho inherente de las personas, enfoca el trabajo conjunto en el establecimiento de políticas públicas sostenibles y costo-efectivas.	Conformación de equipos interdisciplinarios y el trabajo colaborativo. La creación de un espacio permanente de intercambio es fundamental para influir decisiones de alto nivel entre instituciones públicas o privadas del sector salud, las asociaciones profesionales, los profesionales de la salud, colegios y sociedades científicas, y la sociedad civil.

En conclusión, durante la pandemia del COVID-19 el uso de soluciones digitales, principalmente aquellas de uso masivo y bajo costo, ha transformado muchos paradigmas en las relaciones médico-paciente y en la prestación de servicios de salud; No solo mostró el potencial y el impulso que está experimentando la telesalud, sino que dejará muchas buenas prácticas y oportunidades de capacitación, que faciliten el acceso a la salud a los ciudadanos. Sin embargo, es necesario tener en cuenta las limitaciones de la telemedicina y reconocer la importancia del encuentro presencial cuando sea necesario. El eje central de cualquier política de telesalud hoy debe ser el impacto de su implementación para mejorar el acceso a la salud y la calidad de vida del paciente, siempre en condiciones que garanticen la seguridad, confidencialidad y seguridad de su información, y en la continuidad de atención, contribuyendo de esta manera en alcanzar el acceso y cobertura universal en salud de manera costo-efectiva.

## Declaración.

Las opiniones expresadas en este manuscrito son responsabilidad de los autores y no reflejan necesariamente los criterios ni la política de la RPSP/PAJPH o de la OPS.
